# Institutionalising health technology assessment: establishing the Medical Technology Assessment Board in India

**DOI:** 10.1136/bmjgh-2016-000259

**Published:** 2017-06-26

**Authors:** Laura E Downey, Abha Mehndiratta, Ashoo Grover, Vijay Gauba, Kabir Sheikh, Shankar Prinja, Ravinder Singh, Francoise A Cluzeau, Saudamini Dabak, Yot Teerawattananon, Sanjiv Kumar, Soumya Swaminathan

**Affiliations:** 1 Global Health and Development, Imperial College London, London, UK; 2 Indian Council of Medical Research, New Delhi, Delhi, India; 3 Department of Health Research, Ministry of Health and Family Welfare, New Delhi, Delhi, India; 4 Public Health Foundation of India, New Delhi, Delhi, India; 5 PGIMER, Chandigarh, Punjab, India; 6 Health Intervention and Technology Assessment Program, Nonthaburi, Thailand; 7 National Health Systems Resource Centre, Delhi, India

**Keywords:** Health economics, Health policy, Health systems evaluation

## Abstract

India is at crossroads with a commitment by the government to universal health coverage (UHC), driving efficiency and tackling waste across the public healthcare sector. Health technology assessment (HTA) is an important policy reform that can assist policy-makers to tackle inequities and inefficiencies by improving the way in which health resources are allocated towards cost-effective, appropriate and feasible interventions. The equitable and efficient distribution of health budget resources, as well as timely uptake of good value technologies, are critical to strengthen the Indian healthcare system. The government of India is set to establish a Medical Technology Assessment Board to evaluate existing and new health technologies in India, assist choices between comparable technologies for adoption by the healthcare system and improve the way in which priorities for health are set. This initiative aims to introduce a more transparent, inclusive, fair and evidence-based process by which decisions regarding the allocation of health resources are made in India towards the ultimate goal of UHC. In this analysis article, we report on plans and progress of the government of India for the institutionalisation of HTA in the country. Where India is home to one-sixth of the global population, improving the health services that the population receives will have a resounding impact not only for India but also for global health.

**Key messages**

- India is committed to universal health coverage (UHC) for its 1.25 billion population.
- Health technology assessment (HTA) is an important tool to make the priority setting process for allocating health budgets towards UHC more evidence based, fair and equitable.
- The Department of Health Research, Government of India, is taking active measures to formalise a system of HTA in India to form the basis of health policy decisions in the country. Activities to this end are underway and described for the first time in this paper.
- Formalising a system of HTA in India is a promising step in promoting the optimal utilisation of existing resources to ensure that the greatest amount of health is bought for every rupee spent in India and extending adequate healthcare services to the population towards the goal of UHC.

## Introduction

India has experienced steady economic growth over the last decade,^1^ with a consistently growing burden of chronic health problems in line with its growing capital wealth.^2–4^ The rising cost of healthcare has not been matched by a corresponding increase in the healthcare budget, which at 1.3% of gross domestic product (GDP), is one of the lowest in the world.^5^ With the goal of achieving universal health coverage (UHC) and the sustainable development goals in sight,^2 4 6^ decisions on investing in cost-effective health technologies will be critical in improving population health and health equity in India.

Health technology assessment (HTA) provides a globally accepted and structured approach to synthesising evidence for cost and clinical effectiveness alongside ethical and equity considerations to form the basis for evidence-based priority setting and policy decisions.^7 8^ HTA is currently not a formal component of healthcare decision-making in India. Decisions for allocation of health resources at both the national and state level are predominantly made on the basis of consensus opinion from expert committees. However, explicit priority setting is in line with increasing efforts of the Ministry of Health and Family Welfare (MHFW) to strengthen evidence-based service delivery through initiatives such as the institutionalisation of the National Health Accounts^9^ and the development of evidence-based standard treatment guidelines.^10^

Discussion of HTA in India has begun to move beyond academia into official government policy, such as the 12th Five Year Plan^11^ and recently approved National Health Policy,^12^ marking an important shift in the government’s commitment towards a more effective resource allocation for health. To further the HTA agenda in India and implement a formal evidence-based process by which health policy decisions are made in the country, the government of India has assigned the task of establishing the Medical Technology Assessment Board (MTAB) to The Department of Health Research (DHR), a medical research department within the MHFW.

The MTAB will use HTA as a tool for decision-making regarding the allocation of national health resources. Institutionalising HTA in India represents a landmark shift in the way health resource allocation decisions are made in the country, requiring the buy in and cooperation of central, state and local decision makers and from the professional and paraprofessional medical community to maximise effective implementation.

In this paper, we highlight how HTA can assist the Indian government through the MTAB in effective priority setting for UHC; outline a roadmap for effectively embedding HTA in health decision-making and discuss current progress and activities underway, towards achieving this ambitious health sector reform through establishing the MTAB.

## The establishment of MTAB in India

A roadmap for establishing the MTAB and institutionalising HTA has been codesigned by the Government of India, led by the DHR, with technical assistance from national and international research and policy organisations. This roadmap is broadly driven by the core principles laid out in the recent WHO West Pacific Region policy brief ‘Factors Conducive to HTA development in Asia.^13^ The policy brief is the first of its kind to employ a detailed document review, informant interviews, focus group discussions and a survey questionnaire to identify the characteristics of and conducive factors to setting up successful national HTA agencies in the Asia Pacific Region. The study was conducted by a team of scholars from six countries in the Asia Pacific region (China, Indonesia, the Republic of Korea, Malaysia, Thailand and Vietnam), and data informants represented national government, academic institutions, non-governmental organisations and policy think-tanks. The evidence was synthesised and a thematic analysis was conducted to identify common principles that contribute towards successful HTA institutionalisation across Asia. We will outline here each overarching principle for the successful establishment of HTA in an Asian context highlighted by the WHO policy brief, and the corresponding plans for implementation in India through establishment of the MTAB.

### Build human resources and national capacity for HTA

The DHR has recently undertaken a national capacity gap analysis, which was sent to over 50 institutes across country to facilitate understanding of both current capacity to undertake health economic analysis in the country and gauge interest in contributing to the MTAB programme of work. From the results of this survey, memorandums of understanding have been signed between DHR and centres of academic excellence across country to collaborate in this area. A recently published systematic review has highlighted a paucity of direct cost-effectiveness analyses being undertaken and published in India^14^; however, important HTA-related research across the country, including epidemiological, service use and disease burden studies, could contribute as a necessary starting point towards this effort. Capacity building through in-country delivery of intensive health economic training courses, planned to take place in 2017 and 2018, will also provide a necessary basis from which to build local skill and expertise in this area to drive forward the HTA agenda. Future collaboration may be in the form of undertaking analyses, implementation and policy support, providing training or building modules on evidenced-based medicine and priority setting into medical and allied health education programmes.

### Establish a core HTA team or agency

DHR has taken active measures towards hiring an MTAB secretariat as a dedicated workforce to oversee the running of this work programme. This focal HTA agency will be essential to drive the HTA work forward through developing key policy, process and methodology documentation and planning and coordinating all MTAB-related activities.

### Link HTA to policy decision-making mechanisms and processes

The mandate to formally adopt HTA as part of Indian healthcare decision-making, specified in the 12th Five Year Plan^11^ and National Health Policy,^12^ is a positive start to establishing legitimacy and credibility of the MTAB. Initial buy in from policy-makers has been strong, best evidenced by public support of the initiative by both secretaries of state.^15^ Topic nomination and prioritisation will be conducted in consultation with state secretaries of health and the central MHFW to ensure that all future HTA analyses are policy-relevant and user-driven to facilitate immediate uptake into the health system. The way in which recommendations made by the MTAB will be implemented is yet to be fully determined, however, a number of options are presently being explored. The National Health Mission provides a unique portal between the centre and the states through national grants in aid,^9^ where guidance on cost-effective intervention and service delivery guidance could reduce waste and improve allocative efficiency of health spending. The same applies to the national health insurance scheme, Rashtriya Swasthya Bima Yojna, where the MHFW currently lacks evidence to support decision-making regarding cost-effective investment.^16^

### Transparency of HTA agency and processes

MTAB will need to consistently demonstrate its value to both the government and the stakeholder community. Plans for undertaking multiple pilot HTA analyses are underway, where the methods, processes, data availability, technical capacity and evidence to policy continuum will be tested and documented. A conflict of interest policy will be developed to ensure transparency of any pecuniary or non-pecuniary interests of all involved in the analysis and decision-making, based on international best practice standards.^17^ To gain buy in from the stakeholder community, the MTAB must adhere to a standard of full transparency, allowing the public an open window into the process manuals through guiding HTA development, the reference case for economic evaluation, the data and models considered by the MTAB and documentation outlining and potential conflicts of interest and how decisions were ultimately reached.^18 19^ This process of open participation, high technical rigour, and transparency of methods, data, and decision-making, will bring legitimacy and credibility to HTA in India and the MTAB recommendations.

### Take advantage of international collaboration during formative stage of development

The partnership between the government of India and the International Decision Support Initiative, a global network of leading government institutes, universities and think tanks which supports policy-makers in priority setting for UHC, has enabled a demand-driven and contextually appropriate national plan for institutionalising HTA in India, based on a synergy between strong local contextual knowledge and strong international evidence and experience. Successful implementation of HTA in Indian health policy will undoubtedly require an iterative trial and error process, where drawing from the experience of more developed international HTA agenices may also allow potential challenges to be recognised and prospectively addressed. In January 2017, a DHR-led group participated in a study tour to Thailand to understand how HTA has contributed to the country’s achievement of UHC,^20^ providing a promising platform for knowledge exchange and learning. Sustainability requires local ownership, and all parties involved in the early stages of this MTAB programme are committed to ensuring that this process is locally driven, owned and carried forward.

## The promising future of HTA in India

In a health system burdened by rising healthcare costs and inordinately high out of pocket expenditure,^2 5 21 22^ an established and legitimate HTA mechanism in India holds a promising future. The MTAB could provide a platform from which healthcare payers could engage in more strategic purchasing of health services, and offers a crucial mechanism for defining evidence-based criteria for the purchase of services through direct procurement, contracting or health insurance. Further, by specifying appropriate population criteria for which a given HTA recommendation applies, and linking provider payment to fulfilment of these criteria, the government is also afforded a stronger mechanism to enforce regulatory control on service provision and reduce inappropriate delivery of care.^18 23 24^ Such a mechanism not only reduces inappropriate practices and avoids long-term sequelae of inappropriate intervention but also reduces waste of financial resources and the associated opportunity cost.

Adequate public expenditure on health is recognised as an important contributor to the success of embedding HTA in health policy.^13^ While the underlying assumption of HTA is to spend money better, the authors recognise that public health spending in India remains low and can only stretch so far. The recently approved National Health Policy has endorsed the need to raise the level of public spending on health in India to upwards of 2% of GDP.^12^ The existence of an HTA mechanism would allow for well-planned and systematic deployment of newly allocated resources and reduction of potential waste^7 25^ should the government raise public health expenditure in the future.

The complex and fragmented Indian health system architecture poses the most significant challenge to the successful translation of MTAB recommendations for cost-effective service provision translation into practice. Other key challenges relate to health system readiness, data availability and quality, dissemination of information, high out of pocket costs, buy in from the medical and allied health professional communities and the powerful private sector and the availability of mechanisms for monitoring and evaluation.^4 26 27^ The complex relationship between the central and state government also presents an important challenge for the implementation of any federal government initiative, with health as a state-driven subject. In most states, mixed service delivery prevails, in which public sector facilities and programmes deliver many services side by side with expanding social insurance schemes.^27^ HTA needs to acknowledge, address and contribute to improved effectiveness of both private and public care. In the context of national health decision-making, there are key considerations related to the best choice of technologies to ensure that the poor who largely use public facilities receive quality care. However, HTA may also act as a lever to address many of these systemic issues by increasing the credibility, accountability and quality of public and prepaid healthcare services and consequently enhancing their utilisation.^28–30^

## Conclusion

As India strives to deliver UHC, institutionalising HTA to inform how the government invests in health will be increasingly important. HTA will provide a systematic approach to evaluate the properties, effects and costs of health technologies or interventions while considering equity issues and health impact. Institutionalising HTA in India will require government support, technical capability and capacity, awareness among stakeholders, including physicians and allied health professionals, fostering of the culture for the assessment and a fit for purpose health system that can support its implementation uptake of the recommendations from the HTA framework in India. By bringing together Indian academics, health and economic experts and policy-maker representatives, with international experts in the field, the DHR is actively paving a way forward for improving priority setting in India. This represents the beginning of the long path towards building a sustainable HTA framework to inform coverage decisions as part of India’s UHC agenda. In a country that is home to one-sixth of the global population, improving the health of the Indian population will have a resounding impact, not only for India, but for global health in general.
